# Blockage of C-X-C Motif Chemokine Receptor 2 (CXCR2) Suppressed Uric Acid (UA)-Induced Cardiac Remodeling

**DOI:** 10.3389/fphys.2021.700338

**Published:** 2021-07-27

**Authors:** Mingxi Xu, Xu Zheng, Dongxia Wang, Xiaodan Fu, Yida Xing, Yu Liu, Hongjiang Wang, Xiaodan Kong

**Affiliations:** ^1^Department of Rheumatology, The Second Affiliated Hospital of Dalian Medical University, Dalian, China; ^2^Department of Cell Biology, College of Basic Medical Sciences, Dalian Medical University, Dalian, China; ^3^Department of Clinical Laboratory, The Second Affiliated Hospital of Dalian Medicine University, Dalian, China; ^4^Department of Cardiology, The Second Affiliated Hospital of Dalian Medical University, Dalian, China

**Keywords:** CXCL1, CXCL2, uric acid, inflammation, migration

## Abstract

Hyperuricemia-induced cardiac remodeling is at least in part *via* pressure-dependent mechanisms, yet the pressure-independent mechanisms are not well understood. C-X-C motif chemokine ligand 1 (CXCL1) was upregulated in renal tubules from mice subjected to uric acid (UA)-induced nephropathy. Given that CXCL1 is a master chemokine responsible for the recruitment of macrophage by binding with its receptor C-X-C motif chemokine receptor 2 (CXCR2), we thus hypothesized that UA-induced cardiac injury is *via* promoting the recruitment of CXCR2 + macrophages into the heart, which enhances cardiac inflammation. Within a mouse model of UA injection (500 mg/kg, twice/day, 14 days), we measured the level of cardiac CXCL1. We also tested the efficacy of the CXCR2 antagonist on UA-induced cardiac inflammation and remodeling. We found a high plasma level of UA-induced upregulation of CXCL1 in heart tissues. CXCR2 antagonist relieved UA-induced cardiac hypertrophy and suppressed cardiac inflammation and fibrosis. The silencing of CXCR2 in human monocytes abolished the migration of UA-induced monocyte. Thus, the interventions against CXCL1/CXCR2 may be effective for the prevention and treatment of UA-induced cardiac hypertrophy and inflammatory responses.

## Introduction

Uric acid (UA) is the end product of purine metabolism and contributes to the formation of gout (So and Thorens, 2010). Patients with hyperuricemia are at high risk of developing cardiovascular diseases (Tuttle et al., 2001; Siu et al., 2006; Ford et al., 2007). Recently, hyperuricemia is considered as the fourth independent risk factor of heart diseases, besides hypertension, hyperlipidemia, and hyperglycemia (Naithani and Gattner, 1981), reflecting the urgent need for a better understanding of the mechanisms involved.

During inflammatory responses, regional chemokines are elevated and function as a chemoattractant to guide the migration of leukocytes toward the source of the chemokines (Murdoch and Finn, 2000). Based on a shared cysteine motif, chemokines are classified into four distinct classes, such as C-C motif chemokine ligand (CCL1-28), C-X-C motif chemokine ligand (CXCL1-17), C-X3-C motif chemokine ligand 1 (CX3CL1), and X-C motif chemokine ligand 1 (XCL1-2). Chemokines exert their biological functions *via* binding with surface receptors on inflammatory cells, which are designated as C-C motif chemokine receptor (CCR), C-X-C motif chemokine receptor (CXCR), C-X3-C motif chemokine receptor (CX3CR), and XCR chemokine receptor (XCR). CXCL1 is a master factor responsible for the recruitment of macrophages (Remus et al., 2015; Wang et al., 2018; Ntogwa et al., 2020) by binding with its receptor CXCR2. A significant elevation of cardiac CXCL1 is observed in several animal models of heart diseases (Wang et al., 2018). Inactivation of CXCL1 signaling by either a neutralizing antibody or deletion of either CXCL1 or CXCR2 resulted in less macrophage infiltration, reduced cytokine expression level, and preserved cardiac functions following angiotensin II (Ang II) infusion (Wang et al., 2018).

Clinical and experimental studies indicated that the high plasma level of UA was closely associated with renal inflammation and cardiac inflammation (Wijnands et al., 2014; Haryono et al., 2018; Wang et al., 2019; Yang et al., 2019), by promoting leukocyte activation. Within a mouse model of UA-induced nephropathy, CXCL1 was significantly upregulated in renal tubules and promoted inflammatory responses (Wijnands et al., 2014; Haryono et al., 2018; Yang et al., 2019). Given that CXCL1-CXCR2 is involved in cardiac inflammation and remodeling (Wang et al., 2018), we thus hypothesized that UA-induced cardiac injury is *via* the upregulation of CXCL1 in the heart, which enhances macrophage infiltration. Our results provided a novel mechanism for CXCL1-mediated cardiac inflammation in response to UA stimulation. Thus, the interventions against molecular targets such as CXCL1 or CXCR2 may lead to more effective therapeutic strategies for attenuation of UA-induced cardiac inflammatory responses.

## Materials and Methods

### Mouse Hyperuricemia Model

Hyperuricemia was induced by UA injection in Kunming (KM)-background mice (10 weeks, male). UA (500 mg/kg, Sigma) was injected intraperitoneally twice daily for 14 days. The control group only received 0.5% carboxymethyl cellulose sodium (CMC-Na) (in saline) injection for 14 days (Fu et al., 2019). Experimental procedures were approved by the Animal Care and Use Committee of Dalian Medical University. Animal studies are reported in compliance with the Animal Research: Reporting of *In Vivo* Experiments (ARRIVE) guidelines and carried out in accordance with the National Institutes of Health Guide for the Care and Use of Laboratory Animals (NIH Publications No. 8023, revised 1978). Mice were treated with the CXCR2 antagonist [SB265610, dissolved in 2.5% dimethyl sulfoxide (DMSO), 2 mg/g/day, Axon, Groningen, Netherlands], which was administered intraperitoneally 1 day before UA injection for 14 days.

### Echocardiography

Echocardiography was performed on mice with a 30 MHz probe (Vevo 2100, VisualSonics, Canada). Left ventricular (LV) dimensions at end-diastole and end-systole (LVEDd and LVEDs) and anterior wall (Awd/Aws) and posterior wall (Pwd/Pws) thicknesses of end-diastole and end-systole were analyzed. Ejection fraction (EF%) and fractional shortening (FS%) were calculated. Measurements were taken from five consecutive cardiac cycles, and the average was used.

### Blood Pressure Measurement

Blood pressure was measured by the tail-cuff method every 3 days after UA injection.

### Colorimetric Assay for Plasma UA

Mice were anesthetized by 4% chloral hydrate, and blood samples were collected from orbital veins at different time points after UA or vehicle injection. Plasma levels of UA were measured by the Uric Acid Assay Kit (#ab65344, Abcam) according to the instructions of the manufacturer.

### Immunohistochemistry (IHC)

Immunohistochemistry (IHC) was used to determine the contents of CXCL1 or CXCR2 in the LV sections. Briefly, antigen retrieval was conducted by immerging slides in the citrate-ethylenediaminetetraacetic acid (EDTA) buffer and heated in a microwave oven for 5 min. About 10% goat serum was used to block non-specific staining. After blocking, 50 μl of diluted primary antibodies (CXCL1, #ab269939, Abcam; CXCR2, #ab217314, Abcam) was applied onto each section at 4°c overnight. After incubation with biotinylated goat-anti-rabbit secondary antibody (#ab7089, Abcam) for 1 h, sections were incubated with diaminobenzidine (DAB) for 3 min. Sections were counterstained with Mayer’s hematoxylin for 2 min, then dehydrated, then and mounted with DePex.

### Flow Cytometry

Briefly, LVs were minced into multiple small pieces, digested in an enzyme mixture (0.04% collagenase type I and 0.25% trypsin) for 10 min at 37°C, and then stopped by adding 2% fetal bovine serum (FBS). The single cell suspension was resuspended in 100 μl phosphate-buffered saline (PBS) and then incubated with peridinin chlorophyll protein complex (PerCP)-conjugated anti-mouse CD45 antibody (#557235, BD Biosciences), BV421-conjugated anti-mouse CXCR2 antibody (#566622, BD Biosciences), phycoerythrin (PE)-conjugated anti-mouse CD163 antibody (#156704, Biolegend), PE-Cy7-conjugated anti-mouse CD86 antibody (#560852, BD Biosciences), fluorescein isothiocyanate (FITC)-conjugated anti-mouse CD68 antibody (#137006, Biolegend), and allophycocyanin (APC)-conjugated anti-mouse CD11b (#101212, Biolegend) for 30 min in the dark. After staining, samples were analyzed with the FACSCanto II flow cytometer.

### Measuring Cardiomyocyte Size

Alexa Fluor 488 conjugated with wheat germ agglutinin (WGA) (#W11261, 100 μg/ml, Thermo Fisher Scientific) was used to identify individual cardiomyocytes. After 2-h incubation at room temperature, images of five visual fields for each section were acquired across the whole LV with fluorescence microscopy (BX51, Olympus). The cross-sectional area of cardiomyocytes was measured.

### Real-Time PCR

Hearts were collected for gene expression of inflammatory mediators by real-time PCR. The primers used were MCP-1: 5′-CAC TCA CCT GCT GCT ACT CA-3′, 5′-GCT TGG TGA CAA AAA CTA CAG C-3′; IL-8: 5′-CAA GGC TGG TCC ATG CTC C-3′, 5′-TGC TAT CAC TTC CTT TCT GTT GC-3′; CXCL1: 5′-ACT GCA CCC AAA CCG AAG TC-3′, 5′-TGG GGA CAC CTT TTA GCA TCT T-3′; ANF: 5′-CGT CTT GGC CTT TTG GCT TC-3′, 5′-GGT GGT CTA GCA GGT TCT TGA AA-3′; BNP: 5′-GCC ATG TGA GAG TCA GCA AAC-3′, 5′-GTG AGG CCT TGG TCC TTC AA-3′; MHC: 5′-CCT GCG GAA GTC TGA GAA GG-3′, 5′-CTC GGG ACA CGA TCT TGG C-3′; α-SMA: 5′-CCC AGA CAT CAG GGA GTA ATG G-3′, 5′-TCT ATC GGA TAC TTC AGC GTC A-3′; Collagen I: 5′-GCT CCT CTT AGG GGC CAC T-3′, 5′-ATT GGG GAC CCT TAG GCC AT-3′; Collagen III: 5′-TGA CTG TCC CAC GTA AGC AC-3′, 5′-GAG GGC CAT AGC TGA ACT GA-3′; GAPDH: 5′-CAG GAG AGT GTT TCC TCG TCC-3′, 5′-TTT GCC GTG AGT GGA GTC AT-3′. The cycling conditions consisted an initial, single cycle of 5 min at 95°C, followed by 30 cycles of 30 s at 95°C, 30 s at 54°C, and 15 s at 72°C. The gene expression levels were quantified relative to the expression of glyceraldehyde 3-phosphate dehydrogenase (GAPDH).

### Histological Analysis

Hearts were collected from anesthetized mice and then fixed in phosphate-buffered 4% formalin for 24 h, followed by 30% of sucrose for another 24 h. Tissues were embedded in paraffin. Heart histology and fibrosis were stained by H&E and Masson’s trichrome as described earlier. Images were viewed and captured using the microscope (BX51, Olympus).

### Isolation of Neonatal Cardiomyocytes

Hearts from neonatal rats (2–3 days) were isolated and cut into small pieces and then digested in trypsin (Gibco) for 30 min at 37°C. The cell suspension was centrifuged at 1,000 rpm for 5 min and then cultured in Dulbecco’s Modified Eagle Medium (DMEM) supplemented with 10% FBS. After the attachment of fibroblasts (about 2 h), the remaining unattached cells (cardiomyocytes) were collected and then centrifuged at 1,000 rpm for 5 min. The cell pellet was resuspended in DMEM supplemented with 10% FBS.

### Migration Assay

Blood samples (30 ml) were collected from healthy volunteers. Peripheral blood mononuclear cells (PBMCs) were isolated using Ficoll-Paque Plus (Amersham Biosciences, Denmark) according to the instructions of the manufacturer. Monocytes were further sorted by Aria II flow cytometer. Monocytes (1 × 10^7^/ml) were resuspended in Roswell Park Memorial Institute Medium (RPMI 1640) supplemented with 10% FBS (Gibco), penicillin 100 IU/ml, and streptomycin 100 g/ml at 37°C with 5% carbon dioxide (CO_2_). The adenovirus-based small interfering RNA (siRNA) vectors were used for silencing of CXCR2 in monocytes. Neonatal cardiomyocytes were seeded to the lower chamber for 24 h. Cardiomyocytes were stimulated with UA (9 mg/ml) or CMC-Na-saline for another 24 h. About 5 × 10^4^ cells, with or without CXCR2 knockdown, in serum-free RPMI solution (100 μl) were added to the upper chamber of a transwell chamber (Corning). After 12 h, transwell membranes were stained by 4′,6-diamidino-2-phenylindole (DAPI) to detect nuclei. Monocytes accumulating on the lower side of the transwell membrane were acquired by microscopy.

### Statistical Analysis

All data were expressed as mean ± SD. Unpaired t-test and one-way or two-way ANOVA were used to test the mean differences between and within groups if data were determined to be a normal distribution. *P* < 0.05 was considered statistically significant.

## Results

### Uric Acid Injection Induced CXCL1 Upregulation in the Hearts

**FIGURE 1:**
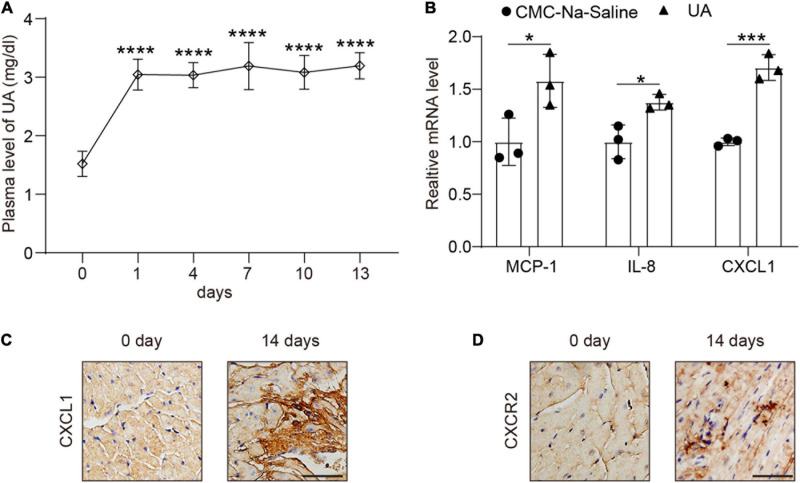
Uric acid (UA) injection induced the upregulation of C-X-C motif chemokine ligand (CXCL1) in the hearts. Plasma levels of UA from mice subjected to carboxymethyl cellulose sodium (CMC-Na) saline or UA for day 14 were measured by ELISA **(A)**. Real-time PCR analysis of the messenger RNA (mRNA) expression of chemokines at day 14 in the same samples **(B)**. Representative images of immunohistochemistry (IHC) staining showing CXCL1 (brown color) accumulation within the hearts from mice subjected to CMC-Na-saline or UA at day 14 **(C)**. Representative images of IHC staining showing C-X-C motif chemokine receptor 2 (CXCR2) (brown color) within the hearts from mice subjected to CMC-Na-saline or UA at day 14 **(D)**. Statistical analysis was performed by one-way ANOVA (Figure 1A) and unpaired t-test (Figure 1B). *P < 0.05, ***P < 0.001, ****P < 0.0001 vs. CMC-Na-saline; *n* = 3/group.

Plasma level of UA was markedly increased at day 1 (Figure 1A) and then kept steady until day 14 (Figure 1A). We then examined the expression level of several chemokines in hearts. CXCL1 had a twofold increase at 14 days after UA injection vs. vehicle control (Figure 1B). The increase in CXCL1 in the heart of the mouse was then verified by IHC (Figure 1C). We then studied whether the rise of cardiac CXCL1 would induce the upregulation of CXCR2 in hearts. Rapid increment of CXCL1 led to the upregulation of CXCR2 at day 14 (Figure 1D).

### C-X-C Motif Chemokine Receptor 2 Antagonist Preserved Cardiac Hypertrophy in Response to UA

**FIGURE 2:**
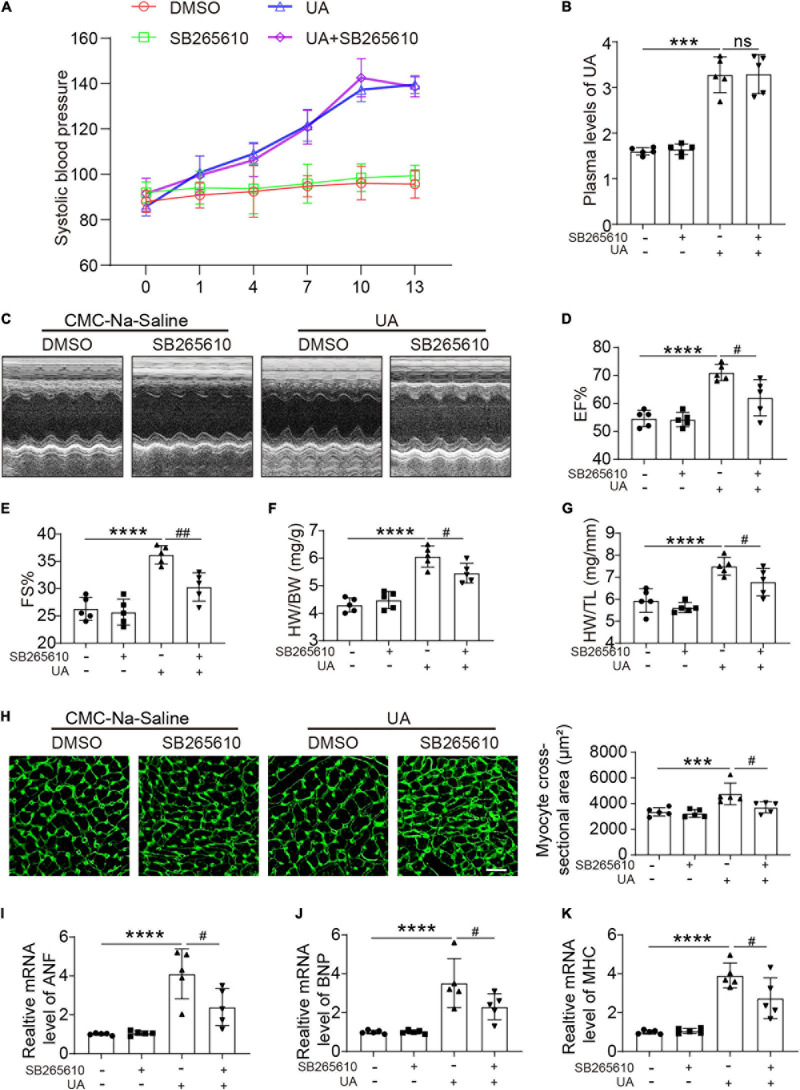
CXCR2 antagonist preserved cardiac dysfunction in response to UA. Systolic blood pressure of mice subjected to CMC-Na-saline or UA, with or without CXCR2 antagonist treatment at day 14 **(A)**. Plasma levels of UA in mice subjected to CMC-Na-saline or UA, with or without CXCR2 antagonist treatment at day 14, were measured by ELISA **(B)**. Representative M-mode echocardiography of left ventricular (LV) chamber **(C)** and echocardiographic assessment of EF% **(D)** and FS% **(E)** from CMC-Na-saline or UA-treated mice, with or without CXCR2 antagonist treatment at day 14. Heart weight/body weight (HW/BW) **(F)** and heart weight/tibia length (HW/TL) ratios **(G)** were analyzed. Heart cross-sectional areas were stained with wheat germ agglutinin (WGA) **(H)**. Real-time PCR analysis of the mRNA expression of hypertrophic markers at day 14 **(I–K)**. Statistical analysis was performed by two-way ANOVA. ***P < 0.001, ****P < 0.0001 vs. CMC-Na-saline + dimethyl sulfoxide (DMSO); ^#^*P* < 0.05, ^##^*P* < 0.01 vs. UA + DMSO; *n* = 5/group; ns: non significance.

C-X-C motif chemokine receptor 2 antagonist had no effect on the blood pressure at day 14 following UA administration (Figure 2A). Elevated plasma UA was also not repressed by the CXCR2 antagonist (Figure 2B). After 14 days of UA injection, we found that EF% and FS% were increased. Inhibition of CXCR2 prevents the enhanced LV contractile function induced by UA, which was measured by EF% and FS% (Figures 2C–E). We further observed that UA-injected mice displayed a marked increase in heart weight/body weight (HW/BW) and heart weight/tibia length (HW/TL) ratios (Figures 2F,G). Such changes were repressed in mice with CXCR2 inhibition (Figures 2C–G). In addition, UA-induced cardiac hypertrophy and hypertrophic markers such as atrial natriuretic peptide (ANP), B-type natriuretic peptide (BNP), and myosin heavy chain (MHC) were upregulated. These changes were also significantly ameliorated by CXCR2 inhibition (Figures 2H–K).

### C-X-C Motif Chemokine Receptor 2 Antagonist Abolished UA-Induced Cardiac Inflammatory Responses and Fibrosis

**FIGURE 3:**
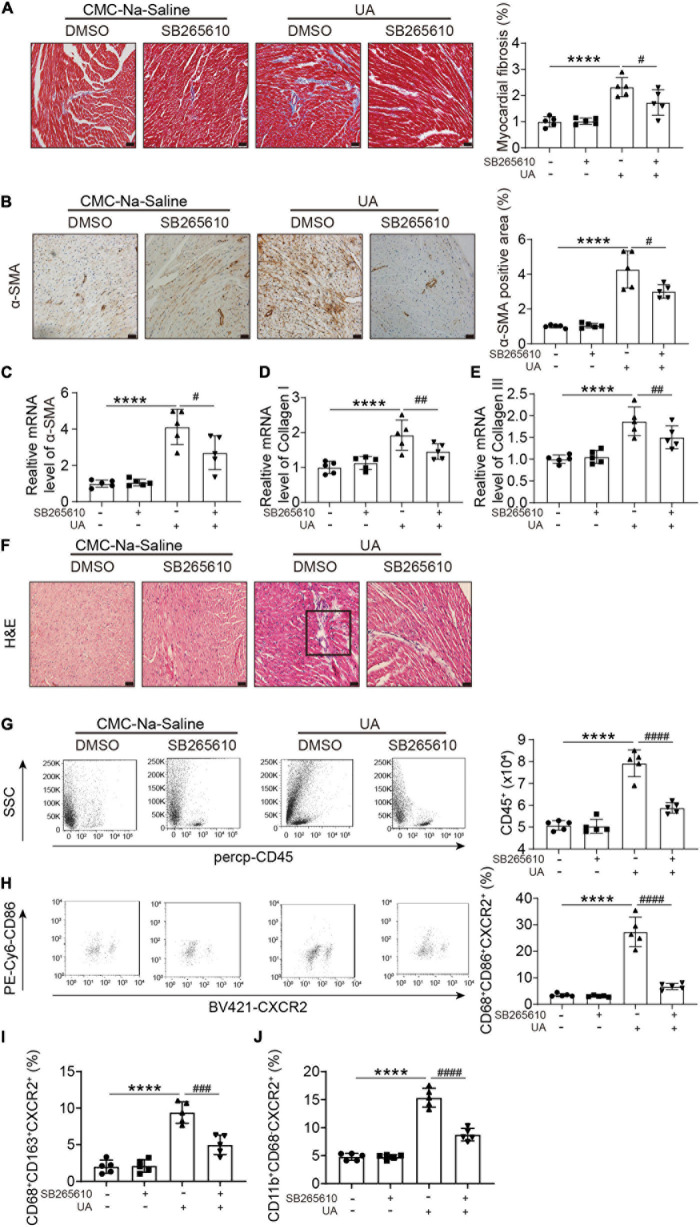
CXCR2 antagonist abolished UA-induced cardiac inflammatory responses and fibrosis. Representative images of Masson’s staining of hearts from mice subjected to CMC-Na-saline or UA, with or without CXCR2 antagonist at day 14 **(A)**. The percentage of the fibrotic area was analyzed in the right panel. Representative images of IHC staining of alpha smooth muscle actin (α-SMA) on hearts sections from mice subjected to CMC-Na-saline or UA, with or without CXCR2 antagonist treatment at day 14 **(B)**. The percentage of positive staining area was analyzed in the right panel. mRNA levels of α-SMA, collagen I, and collagen III in hearts in mice subjected to CMC-Na-saline or UA, with or without CXCR2 antagonist at day 14 **(C–E)**. Representative images of H&E staining of the hearts from mice subjected to CMC-Na-saline or UA, with or without CXCR2 antagonist at day 14 **(F)**. Representative images of flow cytometry displaying infiltrated leukocytes, determined by CD45-positive cells in the hearts from mice subjected to CMC-Na-saline or UA, with or without CXCR2 antagonist treatment **(G)**. Quantitation of infiltrated leukocytes in hearts is shown in the left panel. Representative images of flow cytometry displaying CXCR2^+^ M1 macrophages, determined by CD45^+^/CD68^+^/CD86^+^/CXCR2^+^ cells relative to the whole population of CD45^+^/CD68^+^/CD86^+^ cells in the hearts from mice subjected to CMC-Na-saline or UA, with or without CXCR2 antagonist treatment **(H)**. Quantitation of CXCR2^+^ M2 macrophages, determined by CD45^+^/CD68^+^/CD163^+^/CXCR2^+^ cells relative to the whole population of CD45^+^/CD68^+^/CD163^+^ cells in the hearts from mice subjected to CMC-Na-saline or UA, with or without CXCR2 antagonist treatment **(I)**. Quantitation of CXCR2^+^ neutrophil macrophages, determined by CD45^+^/CD11b^+^/CD68^–^ /CXCR2^+^ cells relative to the whole population of CD45^+^/CD11b^+^/CD68^–^ cells in the hearts from mice subjected to CMC-Na-saline or UA, with or without CXCR2 antagonist treatment **(J)**. Statistical analysis was performed by two-way ANOVA. ****P < 0.0001 vs. CMC-Na-saline + DMSO; ^#^*P* < 0.05, ^##^*P* < 0.01, ^###^*P* < 0.001, ^####^*P* < 0.0001 vs. UA + DMSO; *n* = 5/group. Black square indicates nucleus accumulation.

Uric acid-induced cardiac fibrosis, as measured by Masson’s staining, alpha smooth muscle actin (α-SMA) staining, and expression level of α-SMA, collagen I, and collagen III, was attenuated by the CXCR2 antagonist (Figures 3A–E). H&E staining showed the accumulation of dense nuclei in UA-treated mouse hearts as compared with vehicle control, indicating that inflammatory cells might be recruited to hearts in response to UA stimulation (Figure 3F). This finding was further confirmed by the results from flow cytometry. We found UA-induced severe inflammatory cell infiltration in cardiac (Figure 3G), especially CXCR2^+^ M1 macrophages (Figures 3G,H). These effects were also abolished by CXCR2 antagonist (Figure 3H). Although CXCR2^+^ M2 macrophages and CXCR2^+^ neutrophils were also elevated in response to UA stimulation (Figures 3I,J), the fold increase was less than CXCR2^+^ M1 macrophages.

### Knockdown of CXCR2 Blocked UA-Induced Monocyte Migration *in vitro*

**FIGURE 4:**
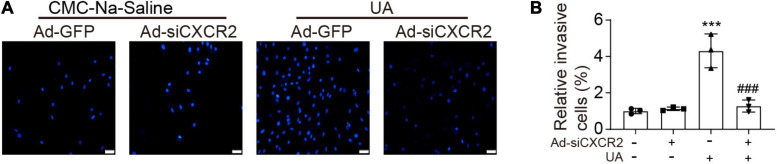
Knockdown of CXCR2 blocked UA-induced monocyte migration *in vitro.* **(A)** Monocytes were loaded to the upper chamber, and cardiomyocytes were cultured in the lower chamber. Migrated monocytes in transwell assays were stained by 4′,6-diamidino-2-phenylindole (DAPI) at 12 h after addition of CMC-Na-saline or UA to the lower chamber. **(B)** Quantitation of migrated cell numbers is presented in the right panel. Statistical analysis was performed by two-way ANOVA. ***P < 0.001 vs. CMC-Na-saline + Ad-GFP; ^###^*P* < 0.001 vs. UA + Ad-siCXCR2; *n* = 3/group.

To test whether UA could directly trigger monocyte migration by activating CXCR2, neonatal cardiomyocytes were cultured in the lower chamber of the transwell and then were stimulated by vehicle or UA for 24 h. Monocytes with or without CXCR2 knockdown were loaded to the upper chamber of the transwell. Compared with the vehicle control group, cardiomyocytes with UA stimulation augmented monocyte migration (Figures 4A,B). Such effects were suppressed by CXCR2 knockdown in monocytes, indicating that UA stimulation leads to the upregulation of CXCL1 in cardiomyocytes, which further recruit monocytes by CXCL1/CXCR2.

## Discussion

In this study, several novel findings have been made. (1) Mice with UA injection upregulated CXCL1 protein levels in hearts; (2) CXCR2 antagonist preserved UA-induced cardiac hypertrophy and suppressed cardiac inflammation and fibrosis; (3) silencing of CXCR2 in human monocytes abolished UA-induced monocyte migration. Thus, the interventions against CXCL1/CXCR2 may be effective for the prevention and treatment of UA-induced cardiac hypertrophy and inflammatory responses.

Clinical and experimental studies reported that high plasma levels of UA induced hypertension and further led to endothelial dysfunction (Khosla et al., 2005; Long et al., 2008; Yang et al., 2019). Clinical studies also revealed that hyperuricemia is closely associated with cardiac dysfunction (Borghi et al., 2015; Zhang et al., 2019), which might be caused by abnormal blood pressure. Wang et al. also demonstrated that CXCR2 mediated infiltration of monocytes into the injured arteries leading to hypertension (Wang et al., 2016). Suppression or knockdown of CXCR2 inhibited Ang II-induced hypertension (Wang et al., 2016). In this study, we confirmed that elevation of plasma UA led to the increase in systolic blood pressure, but inhibition of CXCR2 had no effect on lowering blood pressure. These results indicate that UA induces heart injury and that dysfunction is at least in part *via* a pressure-independent pathway.

In response to various stimulations, cardiac inflammatory responses are upgraded by the infiltration of leukocytes (Prabhu and Frangogiannis, 2016). Chemokines are secreted from cardiomyocytes upon inflammatory stimulation and play an important role in the selective recruitment of leukocytes (Noels et al., 2019). A previous study indicated that CXCL1 was preferable to recruit CXCR2^+^ monocytes to the local sites (Wang et al., 2016, 2018). In this study, we found that CXCL1 was upregulated in UA-injected heart tissues. Further, we confirmed most of the infiltrated macrophages in the heart were CXCR2^+^ macrophages. Monocytes or macrophages display heterogeneity and result in the generation of different subsets that exhibit different functions (Nahrendorf et al., 2007, 2008). M1 subtype is pro-inflammatory, and M2 subtype is reparative. M1 subset expresses tumor necrosis factor alpha (TNF-α), interleukin 1 beta (IL-1β), and matrix metalloproteinases (MMPs), thereby promoting inflammation. In contrast, M2 subset expresses IL-10, TGF-β1, and vascular endothelial growth factor (VEGF), which are necessary for wound healing (Nahrendorf et al., 2007, 2008). In this study, we found most cardiac infiltrated CXCR2^+^ macrophages are M1 subset, indicating that UA-induced cardiac inflammation is largely *via* activation of M1 subset. We further found that deletion of CXCR2 reduced the migrative ability of monocytes, indicating that the recruitment of monocytes is *via* CXCL1/CXCR2.

Mouse models of hyperuricemia are normally induced in two ways (Lu et al., 2019): (1) mice with genetic interventions that result in hyperuricemia (Lu et al., 2018, 2019) and (2) mice that have been exposed to chemical factors (Chen et al., 2015; Wang et al., 2015; Fu et al., 2019). Urate oxidase knockout in C57BL/6J background mice is one of the most commonly used genetic models and had moderately increased serum urate concentrations that are similar to those in humans with hyperuricemia (8.7 ± 2.3 mg/dl in males), associated with multiple organ damages (Lu et al., 2018). Chemical factors, such as uricase inhibitor, Western diet, or direct UA injection, only slightly increased plasma UA concentrations, that is about a twofold increase. In this study, although plasma UA level has not reached the level of human hyperuricemia, we still observe severe cardiac remodeling and hypertension. Our results indicated that patients with increased plasma UA, who even not reached hyperuricemia, still need more attention.

## Conclusion

A high level of UA is able to induce upregulation of chemokine CXCL1 in the heart. Elevated CXCL1 is responsible for attracting CXCR2^+^ macrophages, leading to ventricular hypertrophy. Therefore, inhibition of CXCL1 or CXCR2 would be a potential therapeutic approach against UA-induced heart injury.

## Data Availability Statement

The original contributions presented in the study are included in the article/supplementary material, further inquiries can be directed to the corresponding author.

## Ethics Statement

The studies involving human participants were reviewed and approved by Dalian Medical University Human Research Ethics Committee. The patients/participants provided their written informed consent to participate in this study. The animal study was reviewed and approved by The Animal Care and Use Committee of Dalian Medical University.

## Author Contributions

HW and XK designed the study and wrote the manuscript. MX, XZ, DW, XF, YX, and YL performed the experiments and data analysis. All authors contributed to the revision of the manuscript and approved the final version.

## Conflict of Interest

The authors declare that the research was conducted in the absence of any commercial or financial relationships that could be construed as a potential conflict of interest.

## Publisher’s Note

All claims expressed in this article are solely those of the authors and do not necessarily represent those of their affiliated organizations, or those of the publisher, the editors and the reviewers. Any product that may be evaluated in this article, or claim that may be made by its manufacturer, is not guaranteed or endorsed by the publisher.
